# Dysfunctional Learning and Verbal Memory in Patients with Elevated Tau Protein Levels and Serum Recoverin Autoantibodies—Case Series and Review

**DOI:** 10.3390/brainsci12010015

**Published:** 2021-12-23

**Authors:** Niels Hansen, Claudia Bartels, Kristin Rentzsch, Winfried Stöcker, Dirk Fitzner

**Affiliations:** 1 Department of Psychiatry and Psychotherapy, University Medical Center Göttingen, Von-Siebold-Str. 5, 37075 Goettingen, Germany; claudia.bartels@med.uni-goettingen.de; 2 Euroimmun Reference Laboratory, Seekamp 31, 23650 Luebeck, Germany; k.rentzsch@euroimmun.de (K.R.); w.stoecker@euroimmun.de (W.S.); 3 Department of Neurology, University Medical Center of Goettingen, Robert-Koch Str. 40, 37075 Goettingen, Germany; dirk.fitzner@med.uni-goettingen.de

**Keywords:** recoverin antibody, autoimmunity, cognitive impairment, axonal neurodegeneration

## Abstract

Recoverin-antibody-related disease is currently restricted to late-onset ataxia and autoimmune retinopathy, which can be paraneoplastic or not. However, cognitive dysfunction associated with recoverin antibodies has not been reported so far in a homogeneous patient group. Our case series is dedicated to describing the novel phenotype of cognitive impairment associated with recoverin antibodies. We included five patients with cognitive impairment who presented serum recoverin autoantibodies detected by immunoblots in our case series investigation. We also analyzed their psychopathology, clinical data, cerebrospinal fluid (CSF), and neuroimaging data. Five patients with cognitive impairment associated with serum recoverin antibodies exhibited profound dysfunctional learning and verbal memory. In the CSF of 40% of them, we also diagnosed axonal neurodegeneration entailing elevated tau and phosphorylated tau protein levels. Psychopathologies such as affective symptoms (restlessness, depressive mood, anxiety, complaintiveness) and formal thought disorder, such as rumination, were detected in 25–75% of the patients. We hypothesized a role of recoverin autoimmunity in the pineal gland involving consecutive modulation of hippocampus-based memory caused by an altered release of melatonin. We describe a novel phenotype of possible recoverin autoimmunity in patients with cognitive impairment. However, no clear diagnostic clues can be extracted because of the low diagnostic validity of the testing strategies applied. The possibility of recoverin antibody autoimmunity in the pineal gland correlating with a modulation of hippocampus-based memory should be further investigated.

## 1. Introduction

Recoverin antibodies are known to be associated with both paraneoplastic and non-paraneoplastic retinopathy [1,2,3,4] and late-onset ataxia without retinopathy [5]. However, cognitive dysfunction with recoverin antibodies has not yet been reported in patients. Recoverin is a calcium sensor protein in the nervous system located in the retina and pineal gland [6,7,8,9]. Here we report the novel phenotypic feature of cognitive dysfunction associated with recoverin antibodies.

## 2. Methods

Four patients from the Department of Psychiatry and Psychotherapy of the University Medical Center Göttingen and one patient from the Department of Neurology of the University Medical Center Göttingen were selected because of their cognitive impairment and proven serum anti-recoverin antibodies in immunoblots but not confirmed by a cell-based assay. The patients were screened for antibodies against ANNA-3, Ri, Hu, Yo, Tr/DNER, Ma/Ta, amphiphysin, aquaporin 4, myelin oligodendrocytic glycoprotein (MOG), N-methyl-D-asparate receptor (NMDAR), gamma amino butyric acid B receptor (GABABR), α-amino-3-hydroxy-5-methyl-4-isoxazolepropionic acid receptor (AMPAR), glycine receptor (GlyR), leucin glioma inactivated protein 1 (LGI1), contactin-associated protein-like 2 (CASPR2), IgLON5, recoverin, Zic4, dipeptidyl-peptidase-like protein 6 (DPPX) and myelin. We relied on the Consortium to Establish a Registry for Alzheimer’s Disease (CERAD) to test their neuropsychological performance. Dementia was diagnosed according to the DSM-5 criteria [10], implying dysfunctional higher cortical functions together with a disability in daily living competence. Mild cognitive impairment was present if the daily living competence was not obviously affected, but the patient scored poorly on the CERAD test (performance below −1 z-score). Psychopathology was assessed via the Manual for Assessment and Documentation of Psychopathology in Psychiatry (AMDP) [11]. CSF analysis included cell analysis and the determination of neurodegenerative markers, such as tau protein, phosphorylated tau protein 181, ß-amyloid 42, and ß-amyloid 40. The ß-amyloid 42/40 ratio was determined in the Neurochemistry Laboratory of the Department of Neurology, University Medical Center Göttingen. Cranial magnetic resonance imaging (MRI) was performed either in the Neuroradiology Department of Göttingen or off-site in a nearby neuroradiologic center. We referred to the autoimmune indicators gleaned from the modified criteria published by Hansen et al. [12]. This study was conducted according to the Declaration of Helsinki and was approved by our local ethics committee.

### Statistics

Data are expressed as mean and error of the mean, and percentages calculated from all patients were investigated. Neuropsychological data from the CERAD are shown as z-scores. We considered a neuropsychological performance normal if the z-score was equal to or more than −1.

## 3. Results

### 3.1. Clinical Data

We detected serum antibodies against recoverin in all patients, anti-CV2 in one patient, and anti-Yo antibodies in another patient. One patient revealed CSF antibodies against recoverin. No CSF antibodies were detected in three patients, and none were screened in another patient. The onset age consisted in the mean 69.5 ± 7.5 years (Table 1) with an early onset in two patients. Intrathecal IgG synthesis was present in one of the four patients (25%) (Table 1).

We noted psychopathological deficits (Table 1): deficits in spatial (3/4, 75%) > situational (2/4, 50%) > time orientation (1/4, 25%); deficits in apperception (1/4, 25%), concentration (3/4, 75%), and short- and long-term memory (4/4, 100%); formal thought disorder such as rumination (3/4, 75%), affective dysfunction with depressed mood (1/4, 25%), and anxiety (2/4, 50%); inner restlessness (1/4, 25%); complaintiveness (1/4, 25%); lack of or inhibited drive (2/4, 50%); and social withdrawal (1/4, 25%). Immune anomalies were identified in two patients, one with immunothrombocytopenia and the other with previous breast cancer. Furthermore, we took an extensive differential diagnostic approach in all patients, which included assessing diagnosis-specific antibody indexes for various infectious diseases, such as borreliosis, lues, or viral encephalitis. Metabolic disorders or hypovitaminosis states, such as hypothyroidism or vitamin B12 deficiency, as reasons for cognitive impairment were also excluded. Most importantly, we carefully screened for neurodegenerative disease, as we detected t-tau and p-tau 181 with elevated levels in CSF. As we observed no signs of frontotemporal lobe degeneration, Lewy body disease, rapidly progressing Alzheimer’s disease, Creutzfeldt–Jakob disease, corticobasal degeneration, or any one of these neurodegenerative disorders is unlikely. However, as a limitation we did not seek for genetic abnormalities that might play a role in mild inflammation as recently shown in an animal model to form the basis for encephalitic inflammation [13]. We also found no obvious syndromes or syndromatic constellations that would have justified investigating specific genetic-based syndromes. We identified movement disorder, new-onset headache, paresthesia, tumor, and severe cognitive dysfunction as major autoimmune indicators. cMRI was characterized by focal and general atrophy in 60% of patients. However, unsymptomatic vascular lesions were present in each cMRI as no ischemic attacks or persisting neurological deficits were present. Mean tau protein and phosphorylated tau protein levels were elevated (see Table 1) due to excessive tau and phosphorylated tau protein levels in two of the four patients (50%).

### 3.2. Cognitive Data

**Figure 1:**
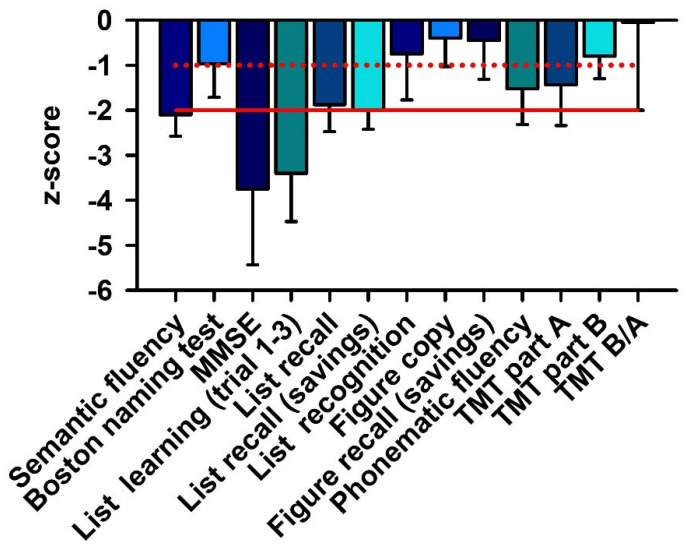
Neuropsychological data.

MMSE revealed a mean test score of 21 ± 4.4, thus reflecting a mild dementia. Three patients revealed mild cognitive impairment, and two patients dementia. Verbal learning and memory are dysfunctional in recoverin-positive patients, who exhibit major impairments in word list learning and minor impairment in word list recall and recognition, but figural memory is unaffected (Figure 1). Semantic and phonematic word fluency and figural memory recall are also slightly affected.

MMSE sum score and learning and memory (list learning, list recall) are severely affected in patients, whereas their neuropsychological performance regarding TMT (information processing, cognitive flexibility) and figural memory is relatively unaffected. The z-score of different cognitive subdomains is depicted. The red line indicates the −2 z-score value, whereas the dotted red line shows the −1 z-score value. A ≤ −2 z-score value is considered a major cognitive dysfunction, and a value ≤−1 a minor cognitive dysfunction. Abbreviations: MMSE = Mini-Mental State Examination, TMT = Trail Making Test.

## 4. Discussion

Our findings suggest a relevant role of recoverin autoimmunity in cognitive dysfunction ranging from MCI to dementia. Our results also argue in favor of recoverin antibodies that might eventually play a role for phenotyping patients in memory clinics. We do not yet know whether recoverin antibodies play a relevant role in inducing cognitive dysfunction, or whether they are an epiphenomenon resembling other antibodies targeting an intracellular antigen, such as GAD65 antibodies.

The relevance of recoverin antibodies in the development of cognitive dysfunction in our case series is unclear, but novel criteria suggest that their cognitive impairments may have an autoimmune origin, as we found indices of specific autoimmune indicators already mentioned, and brain MRIs indicated a chronic degenerative state after long-term inflammation with resulting brain atrophy and, lastly, elevated p-tau 181 and t-tau levels in the CSF as indices of axonal brain damage. Considering all these indices together, we diagnosed a potential autoimmune origin in our patients in line with recently published criteria [12]. Furthermore, to differentiate cognitive impairment associated with recoverin antibodies from other potential causes, we thoroughly considered differential diagnoses, including infectious, metabolic, endocrinologic, or mainly neurodegenerative diseases. To my knowledge, our case series reveals no obvious clinical syndrome, implying an underlying genetic disorder, which is why we conducted no additional genetic screening. Nevertheless, a neurodegenerative disease is also conceivable in some patients presenting no association with detected neural autoantibodies. However, despite this uncertainty, we strongly believe that this report about our cohort is warranted, as their neuropsychiatric presentation is relatively uniform. Moreover, our report may pave the way for a new direction in memory clinics’ research and practice.

Forty percent of our patients presented relevant tauopathy, implying an underlying neurodegeneration that might be the consequence of autoimmune inflammation or even a distinct pathophysiological event. More studies are necessary to investigate the immunopathogenic mechanism behind such tauopathy. As recoverin is mainly expressed in the pineal gland beside the retina, an anti-recoverin autoantibody-associated immunity might be suspected.

The pineal gland’s volume is reduced in Alzheimer’s disease and known to be associated with cognitive impairment [14], suggesting a link between the pineal gland’s volume and its functionality. A reduced pineal gland volume can lower the release of melatonin, thus having a negative impact on neurogenesis and memory [15]. Considering the cognitive dysfunction to be the leading symptom in our patients and their proven serum recoverin antibodies, we speculate that these antibodies targeting recoverin in the pineal gland could affect melatonin release and secondarily lead to impaired neurogenesis and memory function. Melatonin is also known from animal experiments to influence hippocampus-dependent learning in mice [16] and to alter structural synaptic plasticity in granule cells within the dentate gyrus in mice [17]. These animal experiments might demonstrate how pineal autoimmunity caused by recoverin antibodies might modulate hippocampus-dependent learning and the state of memory and its functions.

In addition to the retinopathy known to be associated with recoverin antibodies, a recent report suggested that anti-recoverin antibodies are potentially involved in late-onset ataxia [5]. Whether recoverin plays a specific role in generating ataxia is completely unclear, and it is even less likely than recoverin antibodies being involved in cognitive dysfunction. As cognitive dysfunction is usually not a main symptom of diseases, such as late-onset ataxia and paraneoplastic retinopathy, we feel compelled to report this novel phenotype associated with recoverin antibodies in our homogeneous patient case series. As a study by Dahm et al. [18] showed, recoverin antibodies occur very seldom in neurological and neuropsychiatric diseases. Recoverin autoantibodies were detected in serum in <0.1% in neuropsychiatric and neurological disorders, such as schizophrenia, affective disorders, brain ischemia, Parkinson’s disease, amyotrophic lateral sclerosis, or personality disorder in >2500 patients [18]. We speculate that recoverin autoantibodies might be involved in our patients’ cognitive dysfunction resulting from brain damage, as they revealed indirect indices of brain damage in MRI, such as atrophy and axonal degeneration in their CSF. However, we suggest that known intracellular recoverin autoantibodies per se are not involved in the pathogenesis, but rather that they might trigger T-cell-mediated responses, leading to brain damage. This assumption is derived from knowing that paraneoplastic intracellular antibodies are mainly associated with T-cell-mediated inflammation, as opposed to membrane-surface autoantibodies, which are potentially pathogenic themselves [19]. Furthermore, immune-cell flow cytometric investigations have shown that CD4+ T cells in CSF correlated with cognition in a group presenting cognitive dysfunction and intracellular autoantibodies [20]. Transferring these findings to our case series, we suggest that T-cells are probably playing a role here as sequelae of an immune process within memory-relevant brain structures related to recoverin antibodies, resulting in our patients’ cognitive impairments.

### 4.1. Limitations

As our study sample is too small, no conclusions can yet be made about the diagnostic validity of serum recoverin antibodies in cognitive impairment. Moreover, the immunoblots positive for recoverin could not be confirmed in a cell-based assay, making the diagnostic validity lower than concurring findings from combined diagnostic techniques [21,22]. An immune line blot with Zic4 and Yo positivity together with negative immunohistochemistry has no clear diagnostic significance [22]. However, the same cannot be said about other paraneoplastic antibodies, such as recoverin antibodies, as here a cell-based assay with no immunohistochemistry on rodent brain tissue was used. It is therefore important that recoverin antibody positivity in immunoblots be considered in a clinical data context. Here we describe a unique and uniform clinical presentation involving a cognitive impairment never before reported in relation to recoverin autoimmunity, which deserves attention despite its low diagnostic validity as it probably depicts a part of a recoverin-antibody-positive spectrum disease. We recommend that these patients be followed up to learn how their recoverin autoantibody positivity develops. Another important aspect is that our retrospective case series design did not permit us to apply cutting-edge methods that might have delivered robust evidence of brain damage associated with recoverin autoantibodies. Thus, to keep things simple, we take a somewhat speculative approach in suggesting that the cognitive impairment here may be autoimmune mediated in light of fulfilled criteria from Hansen et al. [12]. In line with this assumption, we postulate that our case series could depict a very mild inflammatory state within an autoimmune continuum involving recoverin autoantibodies. A mild encephalitis is often difficult to detect via standard diagnostics as part of an autoimmune continuum, similar to the hypothesis of a mild encephalitis postulated by Bechter for schizophrenia and bipolar disorder [23] and recently expanded upon for severe mental disorders [24], including cognitive dysfunction. Thus, we believe that detecting recoverin autoantibodies in our patients is not an incidental finding, although that cannot be entirely excluded. Our sample size is too small to draw conclusions for clinical practice, but our pilot data encourage us to investigate recoverin autoantibodies in conjunction with cognitive impairment in a larger patient sample by applying cutting-edge methods, such as flow cell cytometry [25] and volumetric brain neuroimaging of the limbic system [26].

### 4.2. Conclusions

Keeping our small cohort in mind, our case series is nevertheless an initial attempt to characterize a homogeneous patient cohort from a memory clinic who suffered cognitive impairment. Further research is needed in larger patient cohorts to investigate the role of recoverin antibodies in cognitive function and to observe and report on their potential pathogenicity. However, as some patients present indicators of autoimmunity and brain damage, we suggest that such indirect clues imply the pathogenic relevance of these antibodies. Further studies will also have to clarify whether immunotherapy is helpful in this patient cohort.

## Figures and Tables

**Table 1:** Clinical characterization of patients.

PARAMETER	
DEMOGRAPHIC PARAMETER	
Sex (female)	3
Age y	73 ± 2.2
Onset y	69.5 ± 7
Early onset	2/5 (40%)
PSYCHOPATHOLOGY	
Disorders of consciousness	0/4 (0%)
Disturbances of orientation	3/4 (75%)
Disturbances of attention and memory	5/5 (100%)
Formal thought disorder	3/4 (75%)
Worries and compulsions	0/4 (0%)
Delusions	0/4 (0%)
Disorders of perception	0/4 (0%)
Ego disturbances	0/4 (0%)
Disturbances of affect	2/4 (50%)
Disorders of drive and psychomotor activity	2/4 (50%)
Circadian disturbances	0/4 (0%)
Social withdrawal	1/4 (25%)
Excessive social contact	0/4 (0%)
Aggressiveness	0/4 (0%)
Suicidal behavior	0/4 (0%)
Self-harm	0/4 (0%)
Lack of feeling ill	0/4 (0%)
Lack of insight into illness	0/4 (0%)
Uncooperativeness	0/4 (0%)
Need for care	0/4 (0%)
STRONG INDICATORS FOR AUTOIMMUNITY	
Aphasia, mutism, dysarthria	0/5 (0%)
Autonomic disturbances	0/5 (0%)
Central hypoventilation	0/5 (0%)
Decreased level of consciousness	0/5 (0%)
Epileptic seizures	0/5 (0%)
Faciobrachial dystonic seizures	0/5 (0%)
Focal neurological deficit	3/5 (60%)
Hyponatremia	0/5 (0%)
Infectious prodrome	0/5 (0%)
Movement disorder	1/5 (20%)
New onset headache	1/5 (20%)
Adverse response to AP or AD	0/5 (0%)
Optic hallucinations	0/5 (0%)
Other autoimmune disorder	1/5 (20%)
Paresthesia	3/5 (60%)
Presence of a tumor	1/5 (20%)
Presence of neuroleptic malignant syndrome	0/5 (0%)
Severe cognitive dysfunction	5/5 (100%)
WEAK INDICATORS FOR AUTOIMMUNITY	
Confusion	0/5 (0%)
Dynamic course	0/5 (0%)
Early resistance to therapy	0/5 (0%)
Fluctuating psychopathology	0/5 (0%)
CSF	
Cell count (<5 µg/L)	0.4 ± 0.24
Albumin mg/L	247 ± 47
IgG mg/L	23 ± 4.3
IgA mg/L	2.26 ± 0.39
IgM mg/L	0.49 ± 0.14
t-tau protein (<450 pg/mL)	485 ± 174
p-tau 181 (<61 pg/mL)	84.6 ± 38
Aß42 (>450 pg/mL)	948 ± 160
Aß40	11842 ± 807
Aß42/Aß40 ×10 (>0.5)	0.83 ± 0.16
BRAIN MRI	
Generalized atrophy	3/5 (60%)
Focal atrophy	3/5 (60%)
Vascular lesions	5/5 (100%)

Abbreviations: AD = Alzheimer’s disease dementia, CERAD = Consortium to Establish a Registry for Alzheimer’s Disease, CSF = cerebrospinal fluid, GDS = Geriatric Depression Scale, IgA = immunoglobulin A, IgG = immunoglobulin G, IgM = immunoglobulin M, MMSE = Mini-Mental State Examination, MRI = magnetic resonance imaging, NABD = neural autoantibody-associated dementia, p-tau protein 181 = phosphorylated tau protein 181, y = years. The values are depicted as mean ± standard deviation.

## Data Availability

The data are available from the corresponding author.
